# Impact of the COVID-19 pandemic on student teachers: how the shift to online collaborative learning affects student teachers’ learning and future teaching in a Chinese context

**DOI:** 10.1007/s12564-021-09686-w

**Published:** 2021-04-13

**Authors:** Man Lei, Jane Medwell

**Affiliations:** 1 Foreign Languages College, Shanghai Normal University, Shanghai, People’s Republic of China; 2 School of Education, University of Nottingham, Wollaton Road, Nottingham, UK

**Keywords:** Student teachers, COVID-19, Online collaborative learning, Teacher education, China

## Abstract

In March 2020, universities in China transitioned to online education in response to the COVID-19 pandemic and intensified the focus on collaboration in online learning. However, little is known about the impact of undertaking online collaborative learning (OCL) on student teachers’ views about the process and about their own teaching and learning. This qualitative study examined 18 student teachers’ views about their experience of OCL and the way it affected them as learners and future teachers. The participants reported that OCL helped them develop varied views of learning and had a positive effect on their views about the future use of OCL. They saw their personal experience of OCL as an important aspect of their development as teachers. These findings highlight ways that online learning can shape the views and professionalism of student teachers. Future teacher training programs can provide OCL as a teaching experience at an early stage to help transform student teachers’ self-understanding from that of a student to that of a teacher. The findings of this study further reveal that online collaborative teacher training offers student teachers an opportunity to collaborate, discuss, and reflect on their professional development as teachers. This encourages teacher educators to reconsider how new forms of practice and teaching theories can be woven together more effectively in post-COVID teacher training.

## Introduction

The COVID-19 crisis has brought about rapid changes in education worldwide. The consequences of the crisis may take time to become fully apparent, but will potentially have strong impacts (la Velle et al., 2020). In particular, the transfer to online education has challenged teacher education (Murphy, 2020). The need to prepare student teachers to work in complex settings seems to be more crucial than ever as the world faces the current COVID-19 global pandemic (Flores, 2020). In China, universities transferred teacher training exclusively to online delivery. To effectively carry out online education, the Ministry of Education (MoE, 2020) directed university teachers, especially teacher educators, to acquaint students with online learning approaches, and in particular, maximize online collaborative learning (OCL) opportunities for their students. In an attempt to begin to understand the impact of OCL on the next generation of educators, this study addresses the following research question:

- How do student teachers think that their experience of OCL has affected their learning and future teaching?

## Course and adaptations

As part of a full-time four-year undergraduate degree in primary English language education, a cohort of 48 second-year student teachers followed a course focused on language teaching theory and practice. This course was spread over two terms: autumn 2019 (with face-to-face teaching) and spring 2020 (with online teaching). A total of four school visits were initially planned. The first term was teacher-based followed by individual tasks, but no time was devoted to discussions among the students who made two school visits each. The sessions were held twice a week and each session lasted for one and half hours. However, after the face-to-face classes were suspended, the school visits were canceled.

The course was first adapted to asynchronous online instruction that involved the dissemination and storage of session materials on a learning management system with additional annotated PowerPoints and the teacher educator providing voice-over narration. The material for student study was hosted on a learning platform called Xuexitong. However, this online teaching mode spelled a return to a more traditional way of teaching, and as a result of this, the desired levels of learner engagement and outcomes were not achieved. This was further exacerbated by the teacher educator’s lack of experience and skills on online teaching. Consequently, after two sessions, the teacher educator adapted this course to an OCL model that places a great deal with more emphasis on collaborative activities (Magen-Nagar & Shonfeld, 2018).

The OCL project lasted from March 2nd, 2020 to July 8th, 2020. Student teachers were first introduced to OCL (principles, activities, rules, etc.) and then divided into groups. Their subsequent learning was based on online collaborative discussions and product construction, and it included reading, discussions, analysis, and collaborative writing and teaching. Students also used WeChat and Tencent Conference applications. Care was taken to ensure all students were familiar with using these applications. The student teachers were required to follow the following study process:

1. Brainstorming: The student teachers were required to raise ideas and share their thoughts about the teaching behaviors and methods they knew.
2. Identifying, searching for information, and justifying: The student teachers were required to collaboratively contribute to the construction of a shared pool of teaching behaviors to promote learning and justify their choices. The students analyzed techniques and methods from films and materials that the teacher educator shared via the Xuexitong platform. They also searched for information sources by themselves and shared brief descriptions of the teaching behaviors they identified with other groups through the online platform. For example, they selected a scene from a film and added their description of the teaching behaviors and how they promoted students’ learning. The participants were then required to comment on each other’s group works and give credit to the group that posted good work.
3. Comparing and making decisions: After reviewing comments from peers and feedback from the teacher educator, the participants collaboratively constructed a pool of teaching methods. Each group was asked to select at least two teaching methods and jointly write a short paper describing and analyzing the principles as well as the advantages and disadvantages of each approach.
4. Preparing a lesson and conducting teaching and evaluation: Based on the pool of teaching methods they had already created, the participants collaboratively constructed a teaching plan for pupils and explained the teaching approaches they formulated. Students were asked to collect resources themselves and work jointly to record their teaching approaches using online techniques. The participants watched the teaching videos and commented on other groups’ products.

## Literature review

### Online collaborative learning

OCL is one of the accepted teaching approaches in online education. It involves participants working together, exchanging ideas and opinions, developing a shared understanding of specific topics, and constructing collaborative products (Magen-Nagar & Shonfeld, 2018). In OCL models, all learners are considered to be stakeholders and learning takes place through collaborative discourse (Coll et al., 2014). Some scholars assert that OCL is not restricted to discussions and knowledge sharing; instead, it is an effective way for learners to co-construct new knowledge and develop various skills through online collaboration (e.g., Inayat et al., 2013; Margaliot et al., 2018). Previous research suggests that, compared to asynchronous learning experiences, learners can be significantly more active and satisfied with collaborative learning, both in terms of group interaction processes and the quality of group discussions (Ku et al., 2013; Magen-Nagar & Shonfeld, 2018).

Collaborative learning in online courses is generally considered as valuable. OCL enhances interaction between students and teachers and also creates a sense of social presence (Resta & Shonfeld, 2013). This sense can counteract student loneliness when a direct interpersonal touch is missing in distance learning, which can be particularly important during stressful times (Morgan, 2020). This sense also contributes to improving students’ learning and their ability to adapt to various teaching methods, which is in turn beneficial in helping them to understand the complexity of teaching and enhances their motivation and satisfaction (Harasim, 2012). Various researchers have examined participants’ satisfaction with the OCL experience and identified the main influencing factors as including group members’ acquaintance with peers, instructor support and feedback, and reliable and easy-to-use technology (Ku et al., 2013; Magen-Nagar & Shonfeld, 2018). On the other hand, the main source of frustration has been identified as the ability to maintain group participation (Capdeferro & Romero, 2012). OCL can facilitate the development of a more practice-based learning environment for students, which allows participants to learn from their peers, tutors, and on their own. Therefore, OCL can also be considered as an effective way to train future teachers (Anderson, 2020; Margaliot et al., 2018). Recent research has reported the value of practice-based learning networks for student teachers’ sustained professional development in their careers (Margaliot et al., 2018; Park & Son, 2020; Twining et al., 2013). In addition, participating in an online collaborative course develops student teachers’ views of learning and increases student teachers’ knowledge of technology, which may in turn strengthen their positive attitudes toward the use of technology in their own teaching (Hur et al., 2020; Magen-Nagar & Shonfeld, 2018) and lead to enhanced use of OCL in their future teaching. Margaliot et al. (2018) found that student teachers were well versed in technological skills and were well acquainted with using the website, meaning that they were able to work effectively in the online course environment.

### Online teaching during the COVID-19 school closure

There are some emergent studies that support the use of online instruction as an alternative to face-to-face lessons during the COVID-19 pandemic (la Velle et al., 2020; Robinson & Rusznyak, 2020). Scholars and reputable organizations, such as the International Society for Technology (ISTE), have proposed some suggestions for online teaching during this pandemic period. For all students to benefit from online learning, it is important to ensure equity between students, which includes internet access (ISTE, 2020; la Velle et al., 2020; Xue et al., 2020). Moreover, being isolated at home can worsen students’ fears of dealing with the global pandemic. Therefore, responding to students’ emotional issues is particularly important during these stressful times (Anderson, 2020; Tate, 2020), and some of the effective strategies that teachers and teacher educators can use to alleviate students’ fear and anxiety include checking on students regularly, particularly those who are less acquainted with the use of technology (Snelling & Fingal, 2020). Checking on students’ feelings is believed to have a profound effect on their learning (Morgan, 2020). In addition, providing a student-centered learning environment is also necessary during this pandemic. Teachers and teacher educators are encouraged to use a student-centered approach to encourage learners to share ideas, work with peers, and help them at critical points to continue with their online learning (ISTE, 2020; Morgan, 2020). This approach to education changes students’ roles from passively receiving information to actively participating in a process that emphasizes discovery and experience.

Most importantly, it is necessary to explore whether online pedagogies serve the interests of learners and enable the high levels of engagement and outcomes that teachers and teacher educators aspire to achieve. Although there are various forms of online software, applications, and pedagogies, Biesta (2019) expressed concern that online teaching may “spell a return to more traditional ways of teaching” (p. 55). This raises questions as to whether the crisis that we are currently facing might lead us back to traditional ways of teaching or whether new forms of teaching and practice can be more effectively woven together in teacher training. These questions are the focus of the current study. Despite the developing research on OCL and emergent online teaching strategies during the pandemic, there is still little research on student teachers’ perceptions about the effect of the crisis on both their own personal development and their development as student teachers experiencing OCL in their training. Their views are important because they are not only students but also the next generation of teachers.

## Method

The university involved in this study was chosen for convenience because the authors had easy access to the teachers and students there and one of the researchers was the instructor who taught this course. Prior to the data collection procedure, consent forms were distributed to all the student teachers taking the course, and they were assured of their anonymity and confidentiality of the research. The researchers briefly explained the study and emphasized that participation was voluntary. To mitigate the effects of power difference between the researchers and the students, students were informed that the interviews would be conducted after the course had ended (and the instructor/researcher would not be teaching them from the following term). They were also allowed to leave their contact details anonymously if they agreed to participate and assured that their final course grades would not be based on their participation or responses in the interview. Each individual participant was given a code to protect their identity and ensure confidentiality (e.g., ST1 stands for Student Teacher1). The researchers also ensured that the participants would not suffer any disadvantages, violation of privacy, risks as a result of participating in the study, or any emotional harm. The participants also retained the right to withdraw from the research at any stage. A total of 18 participants agreed to take part in the study and were requested to consent to the interviews being recorded. To maintain social distancing, telephone interviews were conducted (in Mandarin) with those who had completed their first module of online learning in July 2020.

The interview questions inquired whether the experience of online teaching and OCL had changed the participants’ views about working online and whether the participants would consider using such techniques in future, as teachers. The questions were piloted and revised with the help of three student teachers who were not subsequently involved in the research.

The interviews lasted between 32 and 60 min, and over the subsequent two months, a clarification of responses (where required) was conducted through either telephone or email. Given that previous research on this area is very scarce, it was difficult for the researchers to predict the responses of the participants. As a result, inductive thematic analysis was employed without any pre-determined set of codes (Braun & Clarke, 2006). For first-level coding, the transcripts were read many times to gain an overall sense of the participants’ perceptions of the impact on their learning and future teaching. Each interview transcript was read as a whole several times. During this process, statements that had the potential to answer the research questions were highlighted (Bogdan & Biklen, 1992). The data were coded into themes that represented the student teachers’ perceptions. In developing the main themes, all the interview transcripts were treated as a whole, without regard to any individual variations. The themes were not meant to describe the variation between individuals, but the range of the themes represented within the interview transcripts as a whole (Marton, 1981). When new themes emerged, previously coded transcripts were revisited to identify any instances of the newly emerging themes that had not been noticed in the initial coding. This whole process was entirely based on the transcripts rather than any preconceptions.

After identifying the statements and coding and categorizing them, the coding was checked as “a good reliability check” (Miles & Huberman, 1994, p.64). Therefore, the themes were repeatedly recategorized and adjustments were made until the themes were stable. During the themes restructuring process, the inter-coder reliability check technique was employed (Maykut & Morehouse, 1994) to enable the researchers to constantly return to previous interviews and compare the themes across cases. This increased the trustworthiness of the final results. To further enhance the trustworthiness of the results, thematic analysis was conducted by two researchers independently, and the main themes surfaced from the aggregated codes.

## Findings

The interview results offer deep insights into how the experience of OCL changed the student teachers’ views about learning and teaching. They identified both aspects of the learning experience and the new skills that they would like to include in their own lives as teachers.

### Aspects of the OCL experience valued by student teachers

Majority of the student teacher respondents (14) considered OCL as a desirable learning experience for them as learners. They identified aspects of their experiences of OCL that they felt had been particularly valuable as counteracting learners’ loneliness, developing a sense of autonomy in them as learners, increasing their self-efficacy, allowing shared rehearsal of learning activities, increasing their motivation for learning, and offering them new insights into teaching methods. Their experience of these aspects of OCL encouraged them to see OCL as a desirable teaching strategy in their own future teaching:

I learned a lot from OCL, and I will definitely use it in my future teaching to help my students advance. (ST2)

The student teachers particularly identified the flexibility of OCL (access to materials, the ability to get feedback online from peers and the teacher, and the opportunity to work at their own pace) as one of its unique features that offer “greater control of learning for students” (e.g., ST4 and ST16).

Four students also identified potential learning as an advantage and highlighted some of the disadvantages of employing OCL in their own teaching in future. Some of the disadvantages identified included: difficulties in developing initial contact between students and challenges maintaining group participation and being able to constructively offer different opinions—“giving face to others is a cultural norm” as one student put it (ST13). Two students specifically related these issues to their own future teaching. They said that they would need to teach their own students how to work in groups and design ways to maintain student understanding about the importance of group work. Student self-regulation was another aspect of OCL that the students believed would be difficult for them to manage as teachers because they had struggled with it themselves:

Sometimes I was frustrated at my inability to maintain self-regulation in OCL. I wanted more of teacher control when I was not familiar with the learning medium. This would be a challenge for me if I used this to teach—letting the students take the lead is not natural for me yet. (ST6)

The student teachers were also concerned about equity for their future students if they employed OCL in their teaching. For instance, ST14 shared the challenges she experienced in accessing the internet and was worried that access would depend on the economic background of the students she was teaching in future. The participants noted that students from low-income families were less likely to have access to the internet and that guaranteeing equity in online access would be a challenge if they adopted OCL in their future teaching.

### How student teachers said OCL had changed their views about learning and future teaching?

The student teachers were asked how they believed OCL had changed their views about learning and teaching. Although the responses were varied, four main themes emerged. Each theme will be outlined below, along with the number of participants whose comments fitted within this theme, to give an indication of the amount of feeling around it. Each theme will then be illustrated using typical student teacher comments.

- Theme 1: OCL changed the student teachers’ views about complex teaching situations (14 commented)

Fourteen student teachers reported that the combination of course assignments and products (e.g., watching films about teacher behaviors and creating a pool of teaching materials) was new to them and had stimulated their interest in working in complex teaching settings:

When we watched the films about how other teachers teach, we discussed the type of behavior that promotes student learning and how. This way, I learned how to deal with particular teaching situations and how to respond in similar settings. (ST4)

ST5 explained that completing course assignments provided an opportunity for her to develop knowledge about how to teach online:

I remember we worked on a topic about teaching methods for e-learning, which I was not familiar with before. Completing this allowed and encouraged me to learn more about the coping strategies to work in complicated online educational settings such as those surrounding the COVID-19 pandemic. This helped me to gain confidence in working in complex situations. (ST5)

For ST2 and ST3, the complexity of their learning was exacerbated by using resources from schools and teachers in various parts of the world. These resources “connected” them to a much wider range of views and helped them stay updated on effective practices that other schools/teachers are using during the COVID-19 pandemic:

The COVID-19 pandemic reminds us that it is more important now than ever to be a connected teacher. Searching resources online helps us actively stay informed about what is new in the world. I will maintain this habit in my future teaching. (ST3)

This suggests that the students were not only relying on the teacher educator as a “resource provider” in OCL but also that the students were transitioning into this role themselves. Relying on themselves to “get to grips with the changes” (ST3) seemed to help the student teachers to develop strategies for experimenting and developing their autonomy and understanding.

Five respondents indicated that they learned to value their own reflections about using OCL through the course. This helped them experience OCL in a supportive and reflective environment:

In the course, our teacher shared with us her own teaching ideas and coping strategies of OCL and guided us to experience it. I learned how to make the theories “realistic” in similar instances, and I could see how I could do this in my teaching. (ST11)

ST8 and ST17 also indicated that the model offered by the teacher, which demonstrated how to use OCL, did not only give them an opportunity to observe their teacher and enhance their pedagogical knowledge, but it also helped them to learn how to internalize and implement theories in their future classroom practice.

Similarly, ST12 also indicated that the emotional support and counseling provided by the teacher educator helped her cope with complex situations and maintain her personal well-being:

I am living in Wuhan, the epicenter of the pandemic, and I suffered strong feelings of worry especially at beginning of this term. However, I received advice and encouragement from my teacher on how to deal with my own problems. This helped me to alleviate my anxiety and I no longer feel isolated. (ST12)

ST12 further note that the teacher showing understanding and care for students is more important than the content that they cover, more so during these stressful times and that she would remember this in her teaching.

Five student teachers mentioned that feedback from their teacher educator and peers had motivated them to consider “what makes a good teacher in times of crisis.” They said this contributed to their personal educational visions:

Our teacher asked us to share our work and comment on each other’s work on Xuexitong. Reading everyone’s comments and watching other peers teaching encouraged me to think about being a good teacher and how to respond in similar crises. (ST1)

- Theme 2: Upgrading their own collaborative learning skills (11 commented)

The participants said that they learned to engage in collaborative work and they valued it. They noted that OCL significantly helped them improve their collaboration skills and decision-making skills. It also enhanced their ability to share work with other group members and voice different opinions:

We faced challenges and disagreements, but this pushed us to explain and share our solutions in various ways to reach a consensus. This way was definitely enlightening and an eye opener because I did not have much collaboration experience before. (ST10)

ST15 viewed the OCL group as a safe space for communication, development of collaborative learning skills, and alleviating anxiety during the COVID-19 pandemic:

Previously, we had no time for any discussions or collaboration. Since the OCL was implemented, we have had more opportunities to discuss and share our ideas. As a result, we are gradually forming a culture of cooperative learning, which supports the development of our collaborative learning skills and, most importantly, alleviates the stress caused by the current crisis since we get to understand that we are not alone. (ST15)

A similar view was expressed by ST7, who emphasized the importance of a “collaborative learning skills set” for learning, future teaching, and professional development:

I hope this kind of “groups” will be retained even after the pandemic because they allow us the space and opportunity to engage in rich conversations about best practices in our learning and teaching. Through them, we can also seek ideas and support from each other, and I see the possibilities of communicating with teachers in other countries. (ST7)

ST8 perceived OCL as a useful technique for improving her teaching skills, and she explained how it helped her improve both her learning and teaching skills:

By experiencing OCL, I have a better understanding of how to plan collaborative work, how to support learners, and what potential challenges they may face. So, I should take these into consideration while planning my teaching. (ST8)

Being involved in OCL as learners also helped the participants to understand the challenges that learners may face and equipped them with skills on how to “organize collaborative tasks more effectively in future teaching” (ST16).

ST3 also indicated that he benefited from activities where collaborators were required to use English to communicate and to create products. His group members made several suggestions on how he could improve his English language proficiency for future teaching.

It is important to note that some feedback from the participants highlighted the challenges they experienced at the beginning of their collaborative learning, especially their unfamiliarity with some group members and the fear that OCL might have a negative impact on their learning. When they started OCL, they were reluctant to leave their comfort zones and felt that they lacked training in collaborative learning skills and online collaboration. Nevertheless, their further explanations suggested that in most cases, they successfully overcame the difficulties they described and learned to value collaboration:

I learned to communicate with others more effectively. We shared good ideas and that made it easy to complete the assignments. (ST10)

It appears that these student teachers were able to assess their communicative skills and learn from others in a positive manner. However, ST18 described her main incentive to develop her collaborative learning skills as “required by the teacher.”

- Theme 3: Developing skills in matching teaching methods to teaching situations (6 commented)

Some participants reported that OCL helped them gain a deeper understanding of the teaching theories that underlie multiple teaching methods. Their experiences also helped them develop flexibility in selecting teaching methods:

We were thinking and planning activities in a way that we would have never thought about before. We learned to compare different teaching methods and choose the most suitable ones for a particular teaching situation. (ST10)

The student teachers indicated that they learned how important it is to have a toolbox of teaching methods. They also reported that they “understood the importance of varying one’s teaching in a manner that connects teaching theories to activities” (ST7). As ST11 reported, the OCL did not only aid her in developing new pedagogical practices and knowledge, but it also helped her develop her self-understanding as a teacher:

Teachers need to stay updated by taking note of any changes in the field and continuing to acquire new pedagogical knowledge and skills. (ST11)

The participants described how the learning process on this course was managed through discovery and practice, which did not solely rely on their tutor teaching them:

We were encouraged to explore and try out our own understanding about teaching. I started to reflect on how I can use the appropriate teaching method to demonstrate my ideas through real practice to help my pupils improve. (ST8)

Such learning not only helped them to transform their mindsets through promoting new ideas about future teaching, but it also assisted them to become more autonomous as professionals.

- Theme 4: Enhancing their technological skills and developing awareness of their responsibility in a digital world (6 commented)

Several student teachers acknowledged that their experiences of OCL had forced them to develop their technological skills and increased their awareness of participation in a digital world. ST1 indicated that:

The COVID-19 pandemic moved us to OCL, and this posed challenges as well as opportunities for us to transform our learning styles, which require us to reorient our views. We should take up the role of being active learners to participate in the digital world, avoid falling behind academically, and work toward being successful teachers. (ST1)

The students appeared to have realized that modern teachers must “constantly develop technological skills to function effortlessly” (ST2) in a world that they are yet to know but in which technological skills remain essential:

Teachers today should commit to upgrading their technological skills continuously with the ultimate goal of benefiting their students and classroom teaching due to the complex, shifting nature of educational settings. (ST2)

ST5 shared her problems in using technologies online and emphasized the importance of checking on students’ feelings regularly as this could have a profound effect on success, especially during this time of crisis:

I am not well conversant with some technologies and this worsened my fear of dealing with the pandemic. Our teacher always checked on my feelings and helped me through. Then I realized that teachers’ simple actions of showing care can have a great impact on students’ learning. (ST5)

ST5’s comment highlights the need for timely technological support for students. With large class sizes and student teachers who are novice users of online education, teachers are likely to experience an unexpected increase in their workloads.

## Discussion

The rapid transfer to online modes of delivery forced the participants to move into a complex educational setting. The student teachers generally showed positive attitudes toward the OCL they undertook and recognized many of its objectives, advantages, and features. However, they also identified aspects of the OCL that were difficult but advantageous as they helped them enhance their skills as learners and future teachers. Their views suggested that some OCL tasks demanded higher levels of autonomy, collaboration, choice, and decision making from learners who were used to a teacher-centered and independent performance-based environment. The participants’ views about learning changed in many ways, which is a clear depiction of what is highlighted in scholarly literature (Hur et al., 2020; Magen-Nagar & Shonfeld, 2018).

The student teachers’ statements about the impact of OCL on themselves show that they realized the added value of this. They were keen to engage in “skills development,” including improving their collaborative learning skills, pedagogical practice and knowledge, teaching techniques, technological skills, and language proficiency to co-construct their new knowledge and develop their own professional expertise. These are the key elements OCL as defined in the studies by Inayat et al. (2013) and Margaliot et al. (2018). Various studies (Hur et al., 2020; Margaliot et al., 2018) have noted that the skill development of participants is the most common element considered in the evaluation of OCL in wider surveys. However, the student teachers in this study went beyond that and recognized the wider role of OCL. They mostly discussed OCL as a learning technique that can prepare them to teach in complex teaching settings, help them to formulate their own educational visions, and enable them to expand their self-understanding as teachers. Therefore, the student teachers in this project viewed OCL as an essential aspect of their sustainable professional development. This reflects a socio-cultural turn in the current teacher education that is impacting upon student teachers’ professional needs. An explanation as to why these findings differ from those of existing studies may simply be the fact that the current COVID-19 pandemic has seen student teachers transition to OCL during a particularly uncertain time, which is inevitably causing them to worry about how they might cope with similar crises in the future. The student teachers in this study repeatedly discussed how OCL had broadened their mindsets, developed their strategies for searching and experimenting changes themselves, and ultimately benefited their future teaching. This suggests a wider view of OCL and hints at a more positive approach to professional change. The student-centered, practice-based nature of the OCL experience appears to have prompted these teachers-to-be to feel that they were “owning” and “constructing” the change rather than passively following it. The challenge that teacher educators and head teachers are facing seems to be to provide prospective teachers with more practice-based and explorative opportunities and to recognize collaborative learning and the development of learning communities as steps toward professionalism and empowerment.

The student teachers in this study reported that they developed flexibility in selecting teaching methods and internalized useful theories about teaching by engaging in not only collaborative assignments and reflections, but also in the observation of their own teacher educator. Recent researchers (Anderson, 2020; Morgan, 2020) point out that one of the ways that student teachers can cope with the complexity of teaching is by learning from their own teacher educators, peers, and practice, which is highly relevant here. Although experience sharing and course videos made by other teachers may be beneficial, students in the present study preferred the content “prepared by [their] own teacher the most” (e.g., ST11). When teacher educators create their own videos and resources, they can also customize the content to ensure the appropriate rigor (Morgan, 2020). By intentionally displaying and sharing ways of employing OCL in practice, teacher educators not only acquaint student teachers with the desirable teaching approaches and techniques that offer them a possible teaching model for the future, but they also influence individual student teachers’ views and their behavior in teaching. Most importantly, the student teachers in this study explained that OCL helps them establish a sense of autonomy, transform their views about teaching, change their behaviors in teaching, and facilitate education innovations in future teaching. This suggests that OCL encouraged the student teachers to take charge of their own professional learning and development. As emphasized in the study by Park and Son (2020), teachers’ autonomous professional development increases their motivation and the tendency to transfer their innovations to their classrooms. To promote the professional development of student teachers, teacher educators are encouraged to help student teachers develop their strategies for sustainable professional development (see Son, 2018, for examples of such strategies).

The student teachers expressed their satisfaction with the teacher educator’s accessibility—in particular, her timely feedback about their work during the course and how she handled their requests. The student teachers’ statements revealed that they could sense that the teacher educator was “approachable” and that her responses at critical points—where problems cropped up—helped the progression of their online learning, which is something that earlier researchers also noted (ISTE, 2020; Morgan, 2020). These studies emphasize the importance of teacher feedback in helping collaborative learning to progress. The present study also found that the student teachers valued the teacher educator’s accessibility in offering them “greater control of learning,” and this helped them to maintain group participation and self-regulation, something not found in previous studies. This can be attributed to the traditional Chinese culture (e.g., teachers showing great control of the class). In addition, the student teachers in this study recognized the added value of the teacher educator’s feedback in motivating them to think about “what a good teacher should do during a crisis,” which also helped them expand their self-understanding as future teachers, something not found in the previous literature.

In addition, the students recognized the emotional support and counseling offered by the teacher educator through the OCL as a crucial factor that had a profound effect on their learning and mental well-being during this time of crisis. Being isolated at home can worsen students’ fears about coping with a global pandemic (Anderson, 2020). The teacher educator in this study checked on the student teachers’ academic progress and feelings regularly, particularly those who were not well conversant with using technology and those who were suffering from anxiety. The students valued the teacher educator’s actions of care as they helped them cope with the complexity of OCL and the stress associated with the pandemic. The educator’s actions are also suggested by Snelling and Fingal (2020) as useful strategies that can be employed by educators during a pandemic. This suggests that teacher educators, as well as teachers, need to put emphasis not only on learners’ learning progress, but also on their personal well-being. Therefore, assigning complex projects requiring materials that are not easily accessible should be avoided and perfection should not be expected.

Majority of the participants in this study (17) recognized their active stance as autonomous learners in OCL. However, comments of ST18 revealed her strong reliance on the teacher educator because she naturally thought of her as a “teacher” in a traditional sense. The power differentials between the teacher educator and the student teachers are to some extent inevitable in Mainland China and even in other similar contexts (e.g., Hong Kong and Taiwan) where the norm is to respect authorities (Lei & Medwell, 2020; Yuan, 2019). However, the comment of ST18 may also suggest that teacher educators need to be aware of these differentials and more importantly, cautious and strategic in guiding their students in different circumstances. In this study, the teacher educator strategically made use of her authoritative stance in scaffolding the student teachers to develop relevant skills and stimulating their continuous learning.

Although majority of the participants (14) were satisfied with OCL, four of them reported that they had a hard time developing initial contacts among groups and maintaining group participation. This finding is corroborated by the findings of Ku et al. (2013) and Magen-Nagar and Shonfeld (2018). Some student teachers were also reluctant in offering different opinions to their peers, findings that are not highlighted in previous studies (Capdeferro & Romero, 2012) but can be attributed to the “cultural norm” in China and other Asian contexts. The student teachers were concerned that these difficulties would impede their learning as the OCL experience forced them to face changes in their learning. For instance, previously, most of their academic assignments had been based on independent learning assignments and were assessed accordingly. However, with the introduction of OCL, they were forced to transition into a collaborative learning environment, with different methods of learning, high levels of autonomy, shared responsibility for learning, and diversity of opinions from group members. In this study, the participants were encouraged to build a collaborative relationship, and they reported that this changed their views about learning. This is corroborated by the findings of earlier studies by Hur et al. (2020) and Magen-Nagar and Shonfeld (2018). They also described OCL as a valuable learning strategy because it counteracted their loneliness and alleviated the anxiety, particularly during this time of a global pandemic (Anderson, 2020). However, since this was the student teachers’ first experience of OCL and they did not have sufficient training or preparation time to familiarize themselves with the method, some of them experienced challenges (e.g., the inability to maintain self-regulation while learning through a medium that they were not used to). Consequently, some claimed that they wanted more “teacher control,” and this reveals their strong reliance on the teacher educator and the need to expand their self-understanding as teachers.

Although OCL increased the participants’ awareness of responsibility in a digital world, their comments about the use of technology suggests that many of them were facing challenges in mastering the technological environment. This finding differs from that of Margaliot et al. (2018), and suggests that educators should not expect all students to master the technological skills of working effectively in online environments. Instead, they should take students’ readiness for online learning into consideration and suggest ways of providing support that can help students take full advantage of online learning opportunities. There are opportunities for teacher educators to enhance their students’ online learning by involving their students in technology integration experiences ranging from lesson planning to the actual implementation of activities (Park & Son, 2020). Furthermore, there are more opportunities for schools to be creative in the use of technological resources in their teaching and practicums.

The findings of this study suggest that ensuring equity is a big challenge for online education. Online education presents some challenges to both teacher educators and student teachers. It requires access to digital technology, such as a computer, and a stable and reliable internet connection. Not all students have their own computers and, therefore, some students may have to share computers with other household members, which is likely to affect their online learning. Many of the teacher students in this study came from low-income households. These students reported that they experienced internet connectivity challenges. To overcome this challenge, schools can provide computers and WiFi hotspot devices to students during the lockdown period, although this would still prove to be a challenge in China due to its large population. Furthermore, increased access to the internet cannot guarantee that equitable services will be provided to all students. For online education to be effective, researchers (Flores, 2020; Morgan, 2020) have suggested that schools need to find ways of communicating their expectations clearly to all the parties involved in the implementation of online education (e.g., teachers, technology service staff, and the students themselves). In this study, the student teachers’ comments revealed that they strongly relied on the teacher educator to solve their technology problems. This implies that the pressure being mounted on teacher educators might be huge. Possible solutions to this challenge include providing a list of frequently asked questions that explain how the school will function during the lockdown to allow staff and students to obtain crucial information and ensuring that students understand what they are supposed to do and who they should ask for help in case they encounter technological problems.

During the first two sessions of the online course, the teacher educator was influenced by the crisis in which she found herself to return to traditional ways of teaching, which corresponds to the findings by Biesta (2019). The first adaptation failed to use online pedagogies to serve the interests of learners and facilitate the high levels of student engagement and outcomes expected; instead, the teacher educator and student teachers engaged in what Biesta (2019, p. 55) described as “a return to more traditional ways of teaching.” Although there is an array of different software and functionalities, as well as some very popular technology-mediated forms of education, online education in real practice can be delivered in more traditional ways that involve “the teacher talking and explaining so that learners can watch, listen and learn” (Biesta, 2019, p. 55). This suggests that teacher educators, school leaders, as well as professional development providers, need to review their approach to online teaching, the goals of that teaching, and its consequences in terms of both teacher educators and student teachers’ engagement. The teacher educator further adapted the course by employing OCL to provide more student-centered and practice-based learning for student teachers—in a way advocated for implementing online learning during a pandemic (ISTE, 2020; Morgan, 2020). As suggested, during the pandemic, instead of using technology to simply present information to students, teachers can provide opportunities for learners to engage in collaborative projects online, use digital tools to collect information, work with peers to create presentations as they share ideas, and allow all voices to be heard, and notably, this seems to have been the case in the OCL experiences of the student teachers in the present study.

## Implications for post-COVID teacher training and conclusion

Although this study focused on student teachers in China, it has wider implications for many researchers, teacher educators, teachers, and professional development providers worldwide. This example from one country shows how online learning can develop the views and professionalism of student teachers, thus empowering them. The findings of this study can help prepare student teachers to face complex education settings and take charge of their professional development. Future teacher training should provide more opportunities for autonomous learning by introducing students to OCL at an early stage in their teacher training. This can help transform their self-understanding from that of being students to that of being future teachers.

This study also offers some important indicators for success in using OCL. Future online courses should share this teaching model’s values, principles, and challenges from the outset to increase students’ skills and motivation. It is also necessary to allow learners some time to enquire and practice. Furthermore, online teacher training courses would benefit from the building of a learning community that begins with self-introduction in groups to reduce the anxiety about distance.

The teacher educator in this study was challenged to develop a new and extended skill set for online teaching. Sharing the success, challenges, and coping strategies of OCL with the student teachers allowed her to model how to cope with a complex teaching setting in a way that was important and realistic to the students. Such sharing can help student teachers to formulate their future teaching visions and enable them to face the challenges of working in times of crisis.

Furthermore, this may not be the last challenge for teacher education, and with the increasing scarcity of placements and school visits, this OCL experience may present an opportunity to reduce placement time for student teachers and still allow them to develop skills that are traditionally delivered through an immersive experience.

The authors hope that the next generation of educators will embrace the concerns mentioned in this study and will be better placed to address them since some new forms of teaching, such as “OCL” and “blended learning,” are likely to remain in place even in post-COVID teaching. This small-scale study has provided examples suggesting that the loss of time on face-to-face lessons can actually be beneficial for student teachers as it gives them more opportunities to collaborate, discuss, and reflect on their professional development as teachers. Professional development providers and teacher educators need to reconsider how new forms of practice and teaching theories can be woven together more effectively.

This study included only 18 student teachers from an undergraduate program on primary English language education in China. Therefore, the generalizability of the findings of this study is limited. Future studies should include more participants from other subject areas and/or national contexts to better understand the similarities and differences among them. However, the findings of this study can offer directions for future research as well as highlight key areas that teacher educators and researchers should consider for improvement. At the time of writing, it has been confirmed that the new term (autumn 2020) will see the integration of OCL and face-to-face sessions. The authors hope that the instructional approaches adopted and challenges faced in this study can help teacher educators prepare effective online or blended instruction. Notably, there is dire need for more research on online teaching and its impact on student teachers’ views, experiences, and future teaching. Although student teachers may be able to demonstrate developing pedagogical knowledge and practice and teaching skills through OCL, this may not fulfill all the requirements to develop as well-rounded, competent teachers. The key competencies missed may need to be re-assessed in later teacher education and future studies. This poses a challenge as well as a possible extension to this study—i.e., examining the key pedagogical knowledge and skills that can be taught and assessed fully via online teacher training.

## References

[CR1] Anderson, L. (2020). ‘Smiles are infectious’: What a school principal in China learned from going remote. *EdSurge*. Retrieved from https://www.edsurge.com/news/2020-03-20-smiles-areinfectious-what-a-school-principal-in-china-learned-fromgoing-remote.

[CR2] Biesta G (2019). Teaching for the possibility of being taught: World-centred education in an age of learning. English E-Journal of the Philosophy of Education.

[CR3] Bogdan RC, Biklen SK (1992). Qualitative research for education: An introduction to theory and methods.

[CR4] Braun V, Clarke V (2006). Using thematic analysis in psychology. Qualitative Research in Psychology.

[CR5] Capdeferro N, Romero M (2012). Are online learners frustrated with collaborative learning experiences?. International Review of Research in Open and Distributed Learning.

[CR6] Coll C, Rochera MJ, de Gispert I (2014). Supporting online collaborative learning in small groups: Teacher feedback on learning content, academic task and social cooperation. Computers & Education.

[CR7] Flores MA (2020). Preparing teachers to teach in complex settings: Opportunities for professional learning and development. European Journal of Teacher Education.

[CR8] Harasim L (2012). Learning theory and online technology: How new technologies are transforming learning opportunities.

[CR9] Hur JW, Shen YW, Cho MH (2020). Impact of intercultural online collaboration project for pre-service teachers. Technology, Pedagogy and Education.

[CR10] Inayat I, Amin RU, Inayat Z, Salim SS (2013). Effects on collaborative web based vocational education and training on learning outcomes. Computers & Education.

[CR11] International Society for Technology in Education (ISTE) (2020). Essential conditions.

[CR12] Ku HY, Tseng HW, Akarasriworn C (2013). Collaboration factors, teamwork satisfaction, and student attitudes toward online collaborative learning. Computers in Human Behavior.

[CR13] la Velle L, Newman S, Montgomery C, Hyatt D (2020). Initial teacher education in England and the Covid-19 pandemic: Challenges and opportunities. Journal of Education for Teaching.

[CR14] Lei M, Medwell J (2020). How do English language teachers understand the idea of professional development in the recent curriculum reforms in China?. Asia Pacific Journal of Education.

[CR15] Magen-Nagar N, Shonfeld M (2018). The impact of an online collaborative learning program on students’ attitude towards technology. Interactive Learning Environments.

[CR16] Margaliot A, Gorev D, Vaisman T (2018). How student teachers describe the online collaborative learning experience and evaluate its contribution to their learning and their future work as teachers. Journal of Digital Learning in Teacher Education.

[CR17] Marton F (1981). Phenomenography: Describing conceptions of the world around us. Instructional Science.

[CR18] Maykut P, Morehouse R (1994). Beginning qualitative research, a philosophic and practical guide.

[CR19] Miles MB, Huberman AM (1994). Qualitative data analysis: An expanded sourcebook.

[CR20] Ministry of Education (MoE). (2020, February). *Notice on supporting education reform and development during the epidemic prevention and control period*. Retrieved from http://www.moe.gov.cn/srcsite/A17/s7059/202002/t20200228_425499.html?from=groupmessage.

[CR21] Morgan H (2020). Best practices for implementing remote learning during a pandemic. The Clearing House: A Journal of Educational Strategies, Issues and Ideas.

[CR22] Murphy MPA (2020). COVID-19 and emergency eLearning: Consequences of the securitization of higher education for post-pandemic pedagogy. Contemporary Security Policy.

[CR23] Park M, Son JB (2020). Pre-service EFL teachers’ readiness in computer-assisted language learning and teaching. Asia Pacific Journal of Education.

[CR24] Resta, P., & Shonfeld, M. (2013). A study of trans-national learning teams in a virtual world. In R. McBride, & M. Searson (Eds.), *Proceedings of the society for information technology and teacher education international conference 2013* (pp. 2932–2940). Chesapeake, VA: AACE.

[CR25] Robinson M, Rusznyak L (2020). Learning to teach without school based experience: Conundrums and possibilities in a South African context. Journal of Education for Teaching.

[CR26] Snelling, J., & D. Fingal. (2020). 10 Strategies for online learning during a coronavirus outbreak. *International Society for Technology in Education*. Retrieved from https://www.iste.org/explore/10-strategies-online-learning-during-coronavirus-outbreak.

[CR27] Son JB (2018). Teacher development in technology-enhanced language teaching.

[CR28] Tate, E. (2020). With weeks of e-learning ahead, be flexible and forget perfection. *EdSurge*. Retrieved from https://www.edsurge.com/news/2020-03-19-with-weeks-of-e-learning-ahead-be-flexible-and-forget-perfection.

[CR29] Twining P, Raffaghelli J, Albion P, Knezek D (2013). Moving education into the digital age: The contribution of teachers’ professional development. Journal of Computer Assisted Learning.

[CR30] Xue E, Li J, Xu L (2020). Online education action for defeating COVID-19 in China: An analysis of the system, mechanism and mode. Educational Philosophy and Theory.

[CR31] Yuan R (2019). A comparative study on language teacher educators’ ideal identities in China: More than just finding a middle ground. Journal of Education for Teaching.

